# Inflammation, HIV, and Immune Quiescence: Leveraging on Immunomodulatory Products to Reduce HIV Susceptibility

**DOI:** 10.1155/2020/8672850

**Published:** 2020-10-27

**Authors:** Ross Cromarty, Derseree Archary

**Affiliations:** ^1^Centre for the AIDS Programme of Research in South Africa (CAPRISA), Nelson Mandela School of Medicine, University of KwaZulu-Natal, Durban, South Africa; ^2^Department of Medical Microbiology, University of KwaZulu-Natal, Durban, South Africa

## Abstract

The relationship between inflammation and HIV has been a focus of research over the last decade. In HIV-infected individuals, increased HIV-associated immune activation significantly correlated to disease progression. While genital inflammation (GI) has been shown to significantly increase the risk of HIV acquisition and transmission, immune correlates for reduced risk remain limited. In certain HIV-exposed seronegative individuals, an immune quiescent phenotype characterized reduced risk. Immune quiescence is defined by specific, targeted, highly regulated immune responses that hinder overt inflammation or immune activation. Targeted management of inflammation, therefore, is a plausible strategy to mitigate HIV risk and slow disease progression. Nonsteroidal anti-inflammatory drugs (NSAIDs) such as hydroxychloroquine and aspirin have shown encouraging preliminary results in low-risk women by reducing systemic and genital immune activation. A topical NSAID, containing ibuprofen, is effective in treating vulvovaginal inflammation. Additionally, the glucocorticoids (GCs), prednisolone, and dexamethasone are used to treat HIV-associated immune activation. Collectively, these data inform on immune-modulating drugs to reduce HIV risk. However, the prolonged use of these pharmaceutical drugs is associated with adverse effects, both systemically and to a lesser extent topically. Natural products with their reduced side effects coupled with anti-inflammatory properties render them viable options. Lactic acid (LA) has immunomodulatory properties. LA regulates the genital microbiome by facilitating the growth of *Lactobacillus* species, while simultaneously limiting bacterial species that cause microbial dysbiosis and GI. Glycerol monolaurate, besides being anti-inflammatory, also inhibited SIV infections in rhesus macaques. The proposed pharmaceutical and natural products could be used in combination with either antiretrovirals for treatment or preexposure prophylaxis for HIV prevention. This review provides a summary on the associations between inflammation, HIV risk, and disease progression. Furthermore, we use the knowledge from immune quiescence to exploit the use of pharmaceutical and natural products as strategic interventions to manage inflammation, toward mitigating HIV infections.

## 1. Introduction

Human immunodeficiency virus (HIV), which causes acquired immunodeficiency syndrome (AIDS), is a global epidemic affecting approximately 37.9 (range 32.7–44) million people with an estimated 1.7 (range 1.4–2.3) million new infections for the year of 2018 [1]. Currently, sub-Saharan Africa (SSA) is the worst affected region with 20.6 (range 18.2–23.2) million infected individuals, with 800,000 new infections in the region during 2018 [1]. Furthermore, young women (15–24 years) are of particular concern in SSA as they account for over half of new HIV infections in this region [2]. The roll out of antiretroviral (ARV) drugs for infected populations has significantly altered the trajectory of the disease and the epidemic, transforming it into a manageable chronic condition for the majority of infected individuals [3]. The use of ARVs as preexposure prophylaxis (PrEP) for prevention has shown promise in men who have sex with men (MSM) populations taking oral PrEP [4–7]. However, variable degrees of success with oral PrEP have been found in heterosexual populations [8–11]. The use of PrEP topically, in formulations such as microbicides gels and vaginal rings among others, has also shown some promise [12, 13]. However, despite the relative success of these PrEP trials, behavioral factors such as PrEP adherence [14] and biological factors such as genital inflammation [15] and a dysbiotic vaginal microbiome [16] have been shown to undermine these prevention strategies [17]. This review summarizes our current knowledge on the interplay between HIV and inflammation and the causes and consequences of inflammation. We also provide insight into immune quiescence as a protective factor against HIV acquisition with special emphasis on the putative use of pharmaceutical or natural products toward inducing a quiescent genital immune environment.

## 2. Inflammation and HIV

Inflammation has been associated with an increased risk of HIV transmission and acquisition [18–21]. In HIV-infected individuals, increased proinflammatory cytokine levels and immune activation directly correlated with increased HIV viral loads in genital secretions [20–23], thereby increasing the probability of onward transmission. Inflammatory cytokines, tumour necrosis factor-*α* (TNF-*α*) and interleukin-1 (IL-1), were shown to directly affect HIV replication by activation of the NF-*κβ* transcription factor, which binds to the HIV promoter region [24]. Furthermore, in a nonhuman primate (NHP) model of simian immunodeficiency virus (SIV) infection, higher monokine induced by interferon-*γ* (MIG) and interferon-*γ*-induced protein 10 (IP-10) mRNA levels in lymph nodes were positively correlated with more rapid disease progression [25]. However, antibody blocking of MIG was shown to reduce HIV-1 replication in an ex vivo human cervical tissue model [26]. In women who were shedding HIV compared to those with undetectable HIV in the genital tract, increased concentrations of TNF-*α*, IL-1*β*, IL-6, and IL-8 were found [27]. Furthermore, monocyte chemoattractant protein 1 (MCP-1) was reported to be positively correlated with viral loads and promoting X4-tropic HIV infection of resting CD4+ T cells [28].

There are various mechanisms whereby inflammation creates a conducive environment for HIV acquisition. In HIV-uninfected individuals, inflammation resulted in recruitment of HIV target cells and epithelial barrier damage [29–31]. Moreover, immune activation and increased cytokine levels were directly associated with increased HIV acquisition risk in both the blood [32, 33] and the genital tract [19, 34]. Nazli et al. [35] demonstrated that mucosal epithelial cells secreted increased proinflammatory cytokines upon exposure to HIV-1. In addition, TNF-*α* and interferon- (IFN-) *γ* have been implicated in reduced epithelial barrier function, thereby increasing permeability of the mucosal barrier [35–40]. Conversely, IL-17 seems to play a critical role in the maintenance of the mucosal barrier [41, 42]. Li et al. [43] described a process of target cell recruitment in rhesus macaques; macrophage inflammatory protein- (MIP-) 3*α* and IL-8 expression recruit plasmacytoid dendritic cells (pDCs) which in turn secrete MIP-1*α* and MIP-1*β*, which recruit CCR5+ cells. Using an in vivo rhesus macaque model, they showed that inflammation and recruitment of target cells to the genital tract were important events for seeding and forming foci of SIV infection following vaginal challenge [43]. A study by Masson et al. [19] showed that elevated genital tract chemotactic cytokines MIP-1*α*, MIP-1*β*, IL-8, and IP-10, which formed part of the definition for genital inflammation, conferred a more than three-fold increased risk for HIV acquisition [19]. Similarly, a follow-up study by Liebenberg et al. [18] comparing plasma and genital tract cytokine levels showed that increased mucosal concentrations of IP-10, MIP-1*β*, IL-8, and monocyte chemoattractant protein- (MCP-) 1 were associated with increased HIV acquisition risk [18]. MIP-3*α* and IL-8 are important chemokines that facilitate infection through their chemotactic activity involved in the recruitment of HIV target cells [44, 45]. Additionally, IP-10, MIP-1*α*, and MIP-1*β* have also been shown to recruit HIV target cells [46–49]. The MIP-1*α*-CCR5 interaction was also shown to activate the JAK/STAT signalling pathway, which is also a key to initiating cellular proliferation [50] and the inflammatory cascade [51, 52].

## 3. Causes of Genital Inflammation

Various biological and behavioral factors have been implicated in causing inflammation in the genital tract. Biological factors include sexually transmitted infections (STIs) and a dysbiotic vaginal microbiome. Risk for HIV acquisition has been associated with preexisting STIs [53–55], likely due to the inflammatory and immune responses against the causative pathogens [56–58]. Furthermore, asymptomatic STIs can further exacerbate inflammation through elevated genital tract inflammatory cytokine profiles and increase the risk for HIV acquisition [59]. Common STIs associated with increased HIV acquisition risk include the herpes simplex virus (HSV) [60, 61], human papillomavirus (HPV) [62, 63], *Neisseria gonorrhoeae* [64], *Chlamydia trachomatis* [65], and *Trichomonas vaginalis* [66, 67].

A dysbiotic vaginal microbiome, commonly referred to as bacterial vaginosis (BV), occurs when there is a shift from a *Lactobacillus* dominant to a non*-Lactobacillus* dominant genital mucosal environment with highly diverse bacterial communities [68]. This dysbiosis often leads to an inflammatory response and subsequent increase in the permeability of the mucosal epithelia [69–71], thus increasing the risk of HIV acquisition [72–76]. Furthermore, a recent study demonstrated the efficacy of the topical 1% tenofovir gel used in the CAPRISA 004 trial as PrEP was undermined in women who had a non*-Lactobacillus*-dominated microbiome [16]. This decreased efficacy was attributed to the direct metabolism of tenofovir (TFV) by *Gardnerella vaginalis* in women with a non*-Lactobacillus*-dominated vaginal microbiome [16, 77].

Vaginal practices have been noted in certain populations of women, which include practices for intimate female hygiene [78] and to enhance sexual pleasure [79]. These practices include washing, douching, and insertion of products, among others [80]. While no studies have been powered to investigate the link between vaginal practices and HIV risk, there is biological plausibility [81]. Studies have shown that women who practice various forms of vaginal hygiene may impact the vaginal microflora [82, 83], which could lead to a dysbiotic microbiome [84, 85] and a subsequent inflammatory response in the genital tract [69, 70, 86]. Furthermore, although there is no direct evidence, inserting products into the vaginal tract is likely to compromise the mucosal barrier through causing microabrasions for easier HIV viral translocation.

Together, STIs, a dysbiotic vaginal microbiome, and vaginal practices have been shown as major factors driving inflammation in the female reproductive tract (FRT). However, these factors alone are not solely responsible for causing genital inflammation, and further studies are warranted to define the complex immunology of this vulnerable site.

## 4. HIV-Exposed Seronegative (HESN)

The risk of HIV infection is heterogenous across a population. Individuals that are continually exposed to HIV without becoming productively infected over a long period of observation are called HIV-exposed seronegative (HESN). HESN individuals display particular immunological phenotypes that have been posited as immune correlates of protection against HIV [87–89]. Genetic polymorphisms, such as mutations in the KIR, HLA, CCR2, CCR5, IRF-3, and CCL3 genes, conferred a significant protection against HIV in certain HESN individuals [90–95]. One particular genetic polymorphism was the delta 32 mutation in the CCR5 encoding region in the genome [96–98]. Other correlates of protection discovered were the presence or induction of particular immune responses [99] of both innate [100–104] and adaptive immunity [105–109], that were able to control acute infection by either neutralizing the virus [110–112] or killing infected cells [113–115] before the formation of foci or viral propagation could occur. An additional correlate for reduced HIV susceptibility in vitro showed significantly greater sterol metabolism, possibility related to the induction of type-1 interferon genes, in peripheral blood mononuclear cells (PBMCs) from HESN individuals compared to healthy controls [116]. These types of immune responses, which are generally triggered through toll-like receptor (TLR) signalling [117], have increased the interest in using TLR agonists as adjuvants in vaccine research [118, 119] including HIV. However, it should be noted that there is heterogeneity between HESN populations as they do not display the same immune correlates of protection, which makes the comparisons across studies difficult. A particular cohort of HESN commercial sex workers (CSWs) from the Pumwani district in Nairobi, Kenya, has been followed and studied extensively since 1984 [120]. Reduced immune activation and inflammation, commonly termed as immune quiescence, was identified in this group of HESNs. The concept of immune quiescence as a correlate of protection for HIV acquisition has largely stemmed from biological and behavioral studies on this particular cohort.

## 5. Immune Quiescence and HIV Risk

The concept of immune quiescence is not only unique in HESNs but has also been observed in sooty mangabeys, the natural host for SIV. Despite these animals being infected with SIV, with high levels of viral replication and depletion of gut CD4+ T cells, sooty mangabeys do not progress to AIDS [121]. Lower levels of systemic and mucosal CD4+CCR5+ T cells [122], reduced type 1 IFN responses [123], lower Th17 cell numbers [124], and better management of immune activation through IL-10 and regulatory T cell (Treg) upregulation [121] attributed to their quiescent state despite ongoing SIV replication and high viral loads.

Multiple studies have reported reduced immune activation in HESN individuals [89, 104, 125–128] underscoring the importance of modulating inflammation and immune activation or having an immune quiescent environment in an effort to minimize the risk of acquiring HIV. Reduced immune activation, defined by lower CD69 expression on CD4+ and CD8+ T cells, was found to be a correlate of protection in HESN CSWs compared to HIV-uninfected CSWs. In addition, the reduced immune activation was associated with increased frequencies of regulatory T cells [129, 130]. Furthermore, low frequencies of CD4+ and CD8+ T cells expressing HLA-DR, CD38, CD70, and Ki67 were reported in a MSM HESN cohort [131]. Similarly, the uninfected partners of serodiscordant couples had reduced expression of the activation markers CD38, HLA-DR, and CCR5 on CD4+ T cells [132, 133], the target cells for HIV infection. Together, these studies reinforce the concept that lower levels of immune activation are associated with reduced HIV acquisition risk. Our in vitro data show that PBMCs stimulated with lipopolysaccharide (LPS) were less susceptible to HIV infection than the unstimulated negative control [134]. LPS stimulation elicited a strong cytokine response with very limited immune activation (defined by CD38 and HLA-DR expression on CD4+ and CD8+ T cells) [134], partially reminiscent of an immune quiescent environment. These data highlight the potential of TLR agonists to induce protective immune responses; however, the continued management of these immune responses will be necessary to avoid overt inflammation.

Furthermore, molecular studies investigating the function of CD4+ T cells from HESN CSWs found lower levels of gene expression in PBMCs and whole blood compared to HIV-uninfected susceptible CSWs [127, 135]. The most under expressed genes identified in HESN CSWs were involved in T cell receptor signalling and host factors required for HIV replication [127, 135]. Despite the difference in cytokine levels being lost after stimulation, unstimulated PBMCs from HESN CSWs were shown to produce lower levels of cytokines compared to PBMCs from HIV-uninfected HESN [127]. This suggests that PBMCs from HESN CSWs are not immunosuppressed, but rather have lower baseline expression of cytokines. Similarly, even after PBMC stimulation, lower levels of IL-17 and IL-22 were still observed in HESN CSWs, suggesting that HESN individuals have a blunted Th17 response [125]. Th17 cells are an important subset of CD4+ T cells and play an important role in homeostasis of mucosal tissues [136–138]. However, despite Th17 cells being essential for mucosal barrier integrity, they are preferentially hijacked for HIV infection [41, 139]. The duality of Th17 cells has been shown to be an important target and is particularly susceptible to infection in a SIV NHP model [140, 141], while remaining susceptible to preferential depletion in HIV-infected individuals [142, 143].

As the majority of HIV infections occur at the FRT, the immune environment within this compartment will be important in determining HIV risk. In the study by Chege et al. [125], cervical mononuclear cells (CMCs) expressed lower IL-17 and IL-22 after stimulation, suggesting that a blunted Th17 response [125] may be protective against HIV infection. Furthermore, reduced expression of pattern recognition receptors (PRRs), and low cytokine production in culture was reported in HESN CMCs [104]. Despite this, these CMCs produced strong antiviral responses after TLR7/8 stimulation, suggesting that cells with immune quiescent phenotypes from HESNs can still elicit protective antiviral responses against HIV [104]. Lajoie et al. [89] investigated differences between HESN CSWs, HIV-infected CSWs, and HIV-uninfected CSWs and found that HESN individuals had lower expression levels of the proinflammatory cytokine IL-1*α* and IFN-*γ*-regulated chemokines MIG and IP-10. Furthermore, the reduced cytokine expression correlated with higher levels of mucosal antiproteases in HESN individuals, suggesting unique expression patterns of mucosal immune mediators, which create an environment less conducive to HIV acquisition [89]. Additionally, MIG and IP-10 bind to CXCR3, which induces the recruitment of activated T cells [89], suggesting that these two chemokines play an important role in modifying HIV risk. These studies describe immune quiescence in the mucosal compartment, which leads to reduced recruitment of HIV target cells, and hence, a reduced risk for HIV infection. Therefore, inducing an immune quiescent mucosal environment is a biologically plausible strategy to reduce risk of HIV infection. We suggest that the use of immunomodulatory products, possibly in combination with ARVs after thorough and rigorous scientific and clinical trial testing, may be an additional strategy to incorporate into the currently limited HIV prevention options.

## 6. Immunomodulatory Products

Since genital inflammation is regarded as a significant risk factor for HIV infection and immune quiescence was attributed as a correlate of protection in certain populations, products that modulate inflammation are attractive additive HIV prevention options. Increased comorbidities in HIV-infected individuals [144, 145] warrant the use of anti-inflammatory therapies to stem HIV-associated inflammation and immune activation [146–150] to ameliorate disease.

### 6.1. Antiretroviral Drugs

Interestingly, ARVs have been associated with reduced immune activation. In a rhesus macaque model of rectal simian human immunodeficiency virus (SHIV) infection, monkeys that were given oral PrEP had reduced levels of cytokines: IL-15, IL-18, and IL-RA [151]. Healthy human participants taking daily PrEP for 30 days had lower systemic CD8 T cell activation (CD38/HLA-DR coexpression) compared to their baseline before PrEP initiation; however, cytokines and other markers of inflammation in the blood were not affected [152]. In high-risk heterosexual HIV serodiscordant African couples, daily oral PrEP also did not modulate HIV-specific immune responses [153]. There may, however, be a heterogenous effect of ARVs on immunity depending on exposure to HIV or HIV infection itself.

### 6.2. Nonsteroidal Anti-Inflammatory Drugs

Nonsteroidal anti-inflammatory drugs (NSAIDs) are the most common anti-inflammatory drugs prescribed for reducing inflammation and through this process, suppress pain [154]. NSAIDs account for approximately 5–10% of prescribed medication each year [155]. The main mechanism for NSAIDs is through the inhibition of the cyclooxygenase (COX) enzymes, which convert arachidonic acid into prostaglandins [156]. Prostaglandins in turn exhibit varied and seemingly opposite functions such as induction and resolution of inflammation [157]. In the following sections, we discuss some commonly used NSAIDs that are used to treat inflammation.

### 6.3. Aspirin

Acetylsalicylic acid (ASA), commonly known as Aspirin®, is a very common NSAID with fairly good safety profiles [158]. Aspirin® is Food and Drug Administration (FDA) in the United States of America approved and is also readily available as an over-the-counter drug. ASA is commonly used to treat headaches [159], to prevent cardiovascular disease [160–162], and to reduce the risk of breast [163] and colorectal cancer [164].

A study of daily oral Aspirin® in low-risk Kenyan women found reduced levels of systemic and mucosal HIV target CD4+ T cells and Th17 cells and reduced systemic inflammatory cytokines [165]. Even in HIV-infected virologically suppressed patients, low-dose Aspirin® was shown to reduce platelet count, T cell, and monocyte activation [166], thereby reducing the risk of non-AIDS-related morbidities, such as cardiovascular diseases [144].

### 6.4. Ibuprofen

Ibuprofen (IBF) is a frequently prescribed and commonly used NSAID with prominent analgesic and antipyretic properties [167, 168]. IBF has similar efficacies as ASA for the treatment of conditions such as headaches [169]. Furthermore, IBF is used for the treatment of various inflammatory, musculoskeletal, and rheumatic disorders [170]. IBF has also been shown to increase the efficacy of nucleoside reverse transcriptase inhibitors (NRTI) by reducing drug transporter proteins, thus limiting the transport of NRTI's out of the cells [171, 172]. IBF is available commercially as a topical anti-inflammatory (Ginenorm® (ibuprofen isobutanolammonium)) as is a vaginal douche. Ginenorm® is effective for treating the inflammatory condition vulvovaginitis and has a superior action compared to other NSAID topical options such as benzydamine [173].

### 6.5. Indomethacin

The NSAID indomethacin is primarily used not only for the treatment of rheumatoid arthritis but also limits HIV replication [174, 175]. In the presence of indomethacin, but not other NSAIDS (Aspirin®, indoprofen, IBF, or naproxen), MT-4 cells (CD4+ T cell line) displayed reduced HIV replication, measured by ELISA as reduced p24 production [174]. Furthermore, indomethacin suppressed HIV replication further when used in combination with an antiviral plant protein called MAP30 [175]. Additionally, similar to IBF, indomethacin improved NRTI efficacy by reducing drug transporter proteins, thus reducing the efflux of these drugs out of cells [171, 172].

### 6.6. Chloroquine and Hydroxychloroquine

The use of chloroquine (CQ) and hydroxychloroquine (HCQ) have been investigated fairly extensively in HIV-infected populations as well. Both of these drugs, taken orally, have been shown to significantly reduce HIV-associated immune activation [147–150]. Furthermore, chloroquine was also shown to directly limit HIV replication and DC-SIGN-mediated HIV viral transfer to CD4+ T cells both in vitro and in vivo [176].

Daily HCQ use was shown to reduce the numbers of circulating CD4+ CCR5+ and Th17 cells, while mucosal Th17 cells expressed lower immune activation markers CCR5 and CD69 [165]. Oral HCQ administration reduced systemic, but not mucosal, IP-10, and IL-2RA [165]. Furthermore, an HCQ vaginal implant was tested in a rabbit and mouse model, and its immunomodulatory effects were tested in the presence of nonoxynol-9- (N9-) induced inflammation. N9, originally designed as a spermicide, was also hypothesized to reduce HIV infection through disrupting the HIV viral membrane. However, N9 increased HIV risk by causing inflammation [177–179]. In contrast, the HCQ implant alone was able to reduce the recruitment of immune cells, improve mucosal epithelial integrity, reduce T cell activation, and reduce inflammatory cytokine production [180] suggesting that an implant containing an anti-inflammatory drug may reduce the risk of HIV infection.

### 6.7. Glucocorticoids

Glucocorticoids (GCs) are also commonly referred to as corticosteroids, although technically GCs are part of the corticosteroid class of drugs. GCs are produced endogenously in the adrenal glands and other tissues [181] as hormonal compounds and are essential to everyday life to regulate and support the physiological process throughout the body [182]. GCs modulate immunity through the interference of gene transcription, resulting in impaired signalling pathways [183, 184], and through nongenomic effects such as interactions with cellular membranes and receptors to initiate or inhibit signalling responses [185]. GCs have also been shown to induce apoptosis of T cells [186], a potential mechanism for immunomodulation. A major hallmark of AIDS is the steady decline in the numbers of CD4+ T cells through a combination of cellular apoptosis, exhaustion, and subsequent immune system dysfunction [187]. Triggering of the GC-induced tumour necrosis factor receptor family-related (GITR) protein has been shown to limit T cell apoptosis, thereby improving immune function, measured by cytokine expression [188], highlighting the potential of GCs to slow HIV disease progression by limiting CD4+ T cell loss. In the following sections, we discuss some commonly used GCs that are used to treat inflammation.

### 6.8. Dexamethasone

Dexamethasone (DEX) is a commonly used GC. DEX has been shown to reduce cytokines associated with a TH1 response, with concomitant increase of cytokines associated with a Th2 response in human PBMCs [189]. Apart from the traditional GC effects on gene transcription, DEX has additional posttranscriptional regulatory effects [190, 191], enhancing its immunomodulatory effects. Furthermore, DEX has also been shown to reduce arachidonic acid derived from the cellular membranes of epithelial cells [192] and suppression of COX-2 and prostaglandin E2 expression [193], highlighting the additional immunomodulatory effects of this drug. Similar to indomethacin, DEX has shown to inhibit HIV replication in an MT-4 cell line, an effect potentiated by concurrent MAP30 treatment [175]. DEX also suppressed the HIV promoter region, thus inhibiting viral transcription and subsequent replication [194]. However, DEX inhibited the killing of HIV-infected CD4+ T cells by macrophages, mediated through antibody-dependent cellular cytotoxicity, in PBMCs from both HIV-infected and uninfected individuals [195], highlighting that DEX, and likely most GCs, can be overtly immunosuppressive and dampen protective responses too.

### 6.9. Betamethasone

Betamethasone (BMS) is another common GC, which is similar to DEX. BMS is commonly used topically, and these topical formulations have been around for years [196]. In a mouse model, topical BMS reduced the expression of IFN-*γ*, IL-1*β*, TNF-*α*, IL-17, IL-22, and IL-13 induced by TLR7/8 stimulation [197]. Similarly, a topical beclomethasone dipropionate inhibited allergen-induced T cell production of IL-3, IL-5, and GM-CSF [198]. Data from our group shows that BMS was potently immunosuppressive in human PBMCs stimulated with TLR agonists LPS, R848, and Pam3CSK4 and the mitogen PHA and even in our unstimulated condition [199]. Furthermore, despite global immunosuppression, BMS significantly reduced HIV-infected CD4+ T cells in the unstimulated and LPS-stimulated conditions, but not in R848, Pam3CSK4, or the PHA conditions [199]. These results suggest that it may be prudent to understand the inflammatory response at the gene transcription level to identify potential drug targets, which lead to the discovery and formulation of appropriate drugs.

### 6.10. Prednisolone

Prednisolone, another common GC, has been used extensively in reducing HIV-associated immune activation to slow the progression to AIDS [200]. The use of prednisolone has been shown to reduce HIV viral loads, chemokine MCP-1 [201], and HIV-associated immune activation [202]. Furthermore, prednisolone slows the loss of CD4+ T cells and inhibits apoptosis of activated CD4+ T cells in ARV-treated patients and during structured therapy interruption [203–205], hindering the progression to AIDS. Conversely, prednisolone treatment in HIV-infected ARV treatment naïve patients showed no effect on disease progression with continued high viral loads despite reduced immune activation, likely due to increased target CD4+ T cells supporting ongoing viral replication [206].

## 7. Natural Compounds

Anti-inflammatory drugs do have unwanted and off-target adverse effects [207, 208]. Chronic use of NSAIDs has adverse effects on the gastrointestinal tract [209, 210], the kidney [211], and the cardiovascular system [212]. Similarly, chronic GC use can increase the risk for cardiovascular [213, 214] and metabolic diseases [215] and neurodegeneration [216]. Although, topical NSAID and GC treatments have dramatically less common adverse effects, systemic effects have been reported with continued use [217, 218], especially in elderly patients [219]. Mucosal surfaces being more permeable than skin are especially susceptible to potential adverse events [220]. Therefore, natural products that may have minimal, if any side effects, either in combination or alone may provide an alternative for certain indications. Three such products are discussed as follows as these have already been formulated for topical use and have shown promising results from in vitro and animal studies.

### 7.1. Vitamin D

Vitamin D deficiency has been associated with a myriad of diseases such as cardiovascular disease, cancers, chronic lung disease, diabetes, and autoimmune diseases in addition to its well-known role in reduced bone homeostasis [221, 222]. Vitamin D has numerous physiological effects on the immune system [223] as its primary active metabolite is a steroid hormone [224]. Supplementation with the active compound of vitamin D, calcitriol, has proven to be effective in preventing both the initiation and progression of various autoimmune diseases in humanized mice models [225–227]. Vitamin D is available as a topical formulation to treat psoriasis [228]. Vitamin D analogues are known to upregulate Th2 and Treg responses and may counter balance against the adverse effects of GCs [229], which cause global immunosuppression. Combination therapies utilising BMS and another vitamin D analogue, calcipotriol (CAL), were shown to be highly and more effective for treating psoriasis than BMS monotherapy alone [230].

Patients with vitamin D deficiency display a similar immune dysfunction profile to that of HIV-infected patients. A hallmark of HIV disease progression is dysregulated immune activation [231]. Since vitamin D has immunoregulatory properties [223], vitamin D supplementation may be a suitable adjunctive therapy to slow disease progression and possibly lower inflammation and immune activation to limit HIV replication. A clinical trial (http://clinicaltrials.gov identifier NCT03426592) is currently in progress to assess the impact of vitamin D supplementation on HIV latency. Furthermore, the association between the use of certain ARVs and reduced vitamin D levels [231] highlights the need for further studies to identify mechanisms for vitamin D depletion in HIV-infected populations on ARVs. These data may be important at a public health level for vitamin D supplementation into ARV regimens in HIV endemic populations.

### 7.2. Glycerol Monolaurate

The most successful non-ARV-based microbicide is glycerol monolaurate (GML), which is also commonly used in cosmetic products. Two studies in SIV rhesus macaque models demonstrate the effect of GML in preventing SIV infection [43, 232]. Two mechanisms of action were identified; first, GML is a fatty acid monoester, which assists with membrane stabilization by blocking bacterial-induced pore formation and T cell activation [233–236]. Second, GML disrupts T cell signalling and function [237] and inhibits cytokine and chemokine production, thereby preventing the recruitment and activation of HIV target cells and important preceding events for establishment of SIV infection [43]. Furthermore, GML was shown to inhibit *Candida* and *Gardnerella vaginalis* in women [238]; the overgrowth of these two microbes is associated with BV [68] and subsequent inflammation of the genital tract [56, 69, 70]. Additionally, GML had no impact on the *Lactobacilli sp*. [238], the bacterial species generally associated with a healthy vaginal microbiome [68]. GML also inactivates HSV-2 [239] and *Chlamydia trachomatis* [240]. GML with its low side effects profile and its ability, at least in preclinical studies, to prevent SIV infections, is an attractive candidate for topical formulation as an HIV prevention modality.

### 7.3. Lactic Acid

Lactic acid (LA) is a naturally occurring compound commonly found in the female genital tract that is produced by *Lactobacillus* species [241, 242]. The amount of LA depends on the dominance of the *Lactobacillus* species. A vaginal microbiome that is dominated by *Lactobacillus* species, with low abundance of microbial diversity, is often termed a “healthy” vaginal microbiome [243]. Research has been focussed on the role that LA plays in the female reproductive tract. Both the L and D isomers of LA have potent anti-inflammatory effects. Both isomers suppressed the expression of inflammatory cytokines IL-1*β*, IL-6, IL-8, TNF-*α*, RANTES, and MIP-3*α* [244]. Simultaneously, increased expression of the anti-inflammatory cytokine IL-1RA from cervicovaginal epithelial cells was observed, even in the presence of TLR stimulation and seminal plasma [244]. Furthermore, LA has been shown to inactivate HIV in vitro, with the L-isoform more potent than the D-isoform, with this effect not solely due to pH [245]. Similarly, this antiviral effect of LA has been shown from clinical samples, whereby cervicovaginal fluid from women with *Lactobacillus*-dominated microbiomes was shown to inactivate HIV ex vivo [246].

Furthermore, topical LA is versatile and is used for the treatment of various skin and oral complications such as acne vulgaris, melanogenesis, and recurrent aphthous ulcerations, respectively [247–250]. An over-the-counter LA containing vaginal douche was assessed for its impact on vaginal microbiota, with adverse findings of 2.6 fold increased risk for acquiring diverse vaginal microbial species through douching with this product during menses [251]. However, the diverse and dysbiotic vaginal microbiome may arise through a combination of douching [83] and menses [252] and may not be the effect of LA itself, as the majority of the women in this study had a *Lactobacillus*-dominant vaginal microbiome at the start of the study [251]. An LA-based vaginal gel is also currently under investigation for its effectiveness in treating BV compared to the current standard-of-care, metronidazole [253]. As there is a high recurrence rate of BV after metronidazole treatment, a *Lactobacillus crispatus* containing vaginal gel used after metronidazole treatment was effective in preventing BV recurrence [254].

### 7.4. Alternative Natural Products

There are many other natural products that could be considered as possible adjunctive therapy due to their anti-inflammatory effects. Curcumin, a curcuminoid contained in turmeric, is one such natural product. Curcumin has shown potent anti-inflammatory and antimicrobial effects [255–258] and antiviral activity against HIV-1 and HSV-2 [258, 259]. Garlic is another such product that has been shown to display anti-inflammatory effects [260–262]. Similarly, consistent with the growing global acknowledgment of medicinal properties of cannabis [263–265], this plant has been shown to have anti-inflammatory properties [266], mainly attributed to the cannabinoid metabolites contained within the plant [264, 267–270]. Cannabis was found to reduce the level of circulating CD16+ monocytes and levels of IP-10, compared to individuals who did not use cannabis [271]. Similarly, heavy cannabis use in HIV-infected individuals was associated with reduced frequencies of activated CD4+ and CD8+ T cells and intermediate and nonclassical monocytes and cytokine producing antigen presenting cells [272], highlighting the immunomodulatory potential of cannabis in preventing inflammation and immune activation. As attractive as these products may be in modulating inflammation (based largely on in vitro data), their safety and side effects have to be rigorously, scientifically tested.

## 8. Conclusion

Genital inflammation significantly modifies the risk for HIV acquisition, although the causes of genital inflammation and exact biological mechanisms need to be further defined. Inflammation leads to the recruitment and activation of CD4+ T cells, which serve as target cells for HIV infection, with a concomitant disruption of the mucosal barrier allowing for easier viral translocation. HIV replicates more efficiently in activated target cells. Conversely, in the era preceding ARVs and PrEP, immune quiescence has been identified as an immune correlate of protection against HIV infection in some high-risk populations. The use of anti-inflammatories to reduce HIV transmission is therefore not a new concept. Dampening inflammation to induce an immune quiescent phenotype in high-risk populations is attractive as adjunctive therapy in combination with PrEP or in areas where PrEP access is limited. Therefore, the purpose of this review was to highlight the associations between inflammation and increased HIV risk and immune quiescence and HIV and to propose products that may be used to induce immune quiescence to reduce the risk of HIV acquisition. Many pharmaceutical anti-inflammatory drugs have known adverse effects; therefore, we also proposed natural products that may be used either in combination or alone to mitigate HIV risk by reducing genital inflammation. However, inflammation is a necessary and protective response against invading pathogens and damaged tissues. The modulation of specific immune responses that initiate and drive the inflammatory cascade may be a key in preserving a certain threshold of inflammation that is protective. Therefore, interrogating the cellular transcriptional signalling pathways during inflammation will be an important first step in understanding which immunomodulatory products would be appropriate to use to mitigate overt inflammation, while allowing protective inflammatory responses to continue.
